# A re-tightening suture technique for hemostasis during aortic root remodeling in chronic DeBakey type II aortic dissection

**DOI:** 10.1093/jscr/rjag954

**Published:** 2026-10-27

**Authors:** Atsutaka Aratame, Takashi Kunihara, Daisuke Kaku, Toshio Baba

**Affiliations:** Department of Cardiovascular Surgery, Bell-Land General Hospital, 500-3 Higashiyama, Naka-ku, Sakai, Osaka 599-8247, Japan; Department of Cardiac Surgery, The Jikei University School of Medicine, 3-25-8 Nishishinbashi, Minato-ku, Tokyo 105-8461, Japan; Department of Cardiovascular Surgery, Bell-Land General Hospital, 500-3 Higashiyama, Naka-ku, Sakai, Osaka 599-8247, Japan; Department of Cardiovascular Surgery, Bell-Land General Hospital, 500-3 Higashiyama, Naka-ku, Sakai, Osaka 599-8247, Japan

**Keywords:** chronic DeBakey type II aortic dissection, aortic root remodeling, valve-sparing root replacement, re-tightening suture technique

## Abstract

We report a 45-year-old man with chronic DeBakey type II aortic dissection, a sinus of Valsalva aneurysm, and severe aortic regurgitation. His atopic dermatitis remained poorly controlled despite topical corticosteroids and injectable anti-inflammatory therapy and he occasionally experienced cutaneous bleeding from unconscious scratching during sleep. He therefore wished to avoid long-term anticoagulation. Valve-sparing root replacement using root remodeling with external annuloplasty was performed. Bleeding from the proximal continuous suture line was controlled using a re-tightening suture technique that lifted the bleeding point, facilitated placement of an additional mattress suture, and secured the redundant continuous suture under renewed tension. Postoperative imaging showed satisfactory root geometry and no residual aortic regurgitation. This technique may provide a simple and reproducible method for controlling difficult suture-line bleeding during root remodeling.

## Introduction

Valve-sparing root replacement (VSRR) has achieved favorable outcomes in selected patients with acute aortic dissection [1]. However, reports of VSRR for chronic aortic dissection remain limited, and chronic DeBakey type II dissection localized to the aortic root is particularly rare.

We report a case of chronic root dissection with a sinus of Valsalva aneurysm and severe aortic regurgitation successfully treated using root remodeling and a re-tightening suture technique.

## Case report

A 45-year-old man presented with severe aortic regurgitation and a 60-mm sinus of Valsalva aneurysm caused by chronic DeBakey type II aortic dissection (Fig. 1A–C). The dissection was predominantly confined to the aortic root, without a clearly mobile intimal flap.

**Figure 1:**
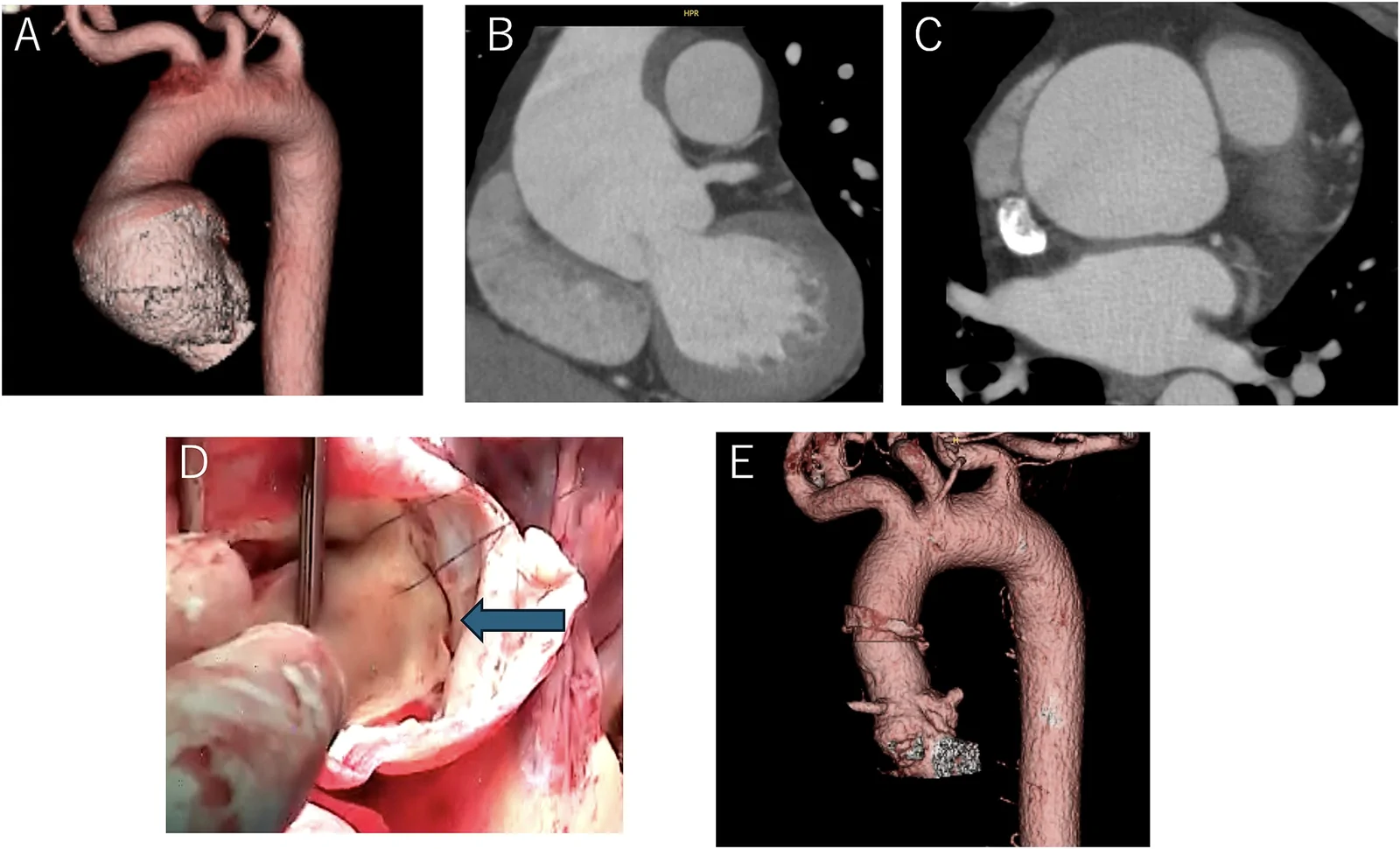
Preoperative contrast-enhanced computed tomography images (A–C); the aneurysm had an asymmetrical morphology without a clearly visible intimal flap (A and B); the maximum short-axis diameter of the sinus of Valsalva aneurysm was 60 mm (C); intraoperative view showing a scarred tear above the sinotubular junction (arrow; D); postoperative contrast-enhanced computed tomography demonstrating satisfactory reconstructed root geometry (E).

The patient had poorly controlled atopic dermatitis despite treatment with topical corticosteroids and injectable anti-inflammatory therapy. He sometimes unconsciously scratched his skin during sleep, resulting in skin bleeding. He was concerned that long-term anticoagulation could exacerbate these episodes and strongly wished to avoid a procedure involving a mechanical valve. Because the cusps appeared suitable for preservation, VSRR was selected through shared decision-making. The regurgitation was considered mainly functional and attributable to sinotubular junction dilatation. Root remodeling was selected because dense adhesions from chronic dissection were anticipated and extensive root dissection was considered undesirable.

Surgery was performed through a median sternotomy. Dense inflammatory adhesions surrounded the ascending aorta and aortic root. A scarred tear consistent with previous dissection was identified above the sinotubular junction and was considered responsible for root dilatation (Fig. 1D). The basal ring measured 28 mm. Cusp geometric heights and free-margin lengths indicated that valve preservation was feasible.

A 24-mm vascular graft was selected from the geometric measurements. Three symmetric tongues were created using a 24-mm SK-I template (a custom-designed template for aortic valve repair) and sutured to the native root with continuous 4–0 polypropylene sutures. The commissures were reconstructed at equal heights. External annuloplasty was performed using an expanded polytetrafluoroethylene suture tied over a 22-mm Hegar dilator. Because the effective height of the right coronary cusp was 7 mm, compared with 10 mm for the other cusps, central plication was added to the right coronary cusp. Aortoscopy confirmed adequate cusp height and coaptation.

Before restoration of cardiac activity, the graft was filled with blood to assess hemostasis, revealing bleeding from the proximal continuous suture line at the reconstructed sinuses. At each bleeding point, a separate suture was passed around the continuous suture line and lifted to expose the site. An additional horizontal mattress suture was then placed. The redundant segment of the continuous suture was pulled and secured within the additional mattress suture, thereby re-tightening the original suture line (Fig. 2 and Video 1). The coronary arteries were reconstructed using the inclusion technique, and the same re-tightening maneuver was applied around the coronary sites.

**Figure 2:**
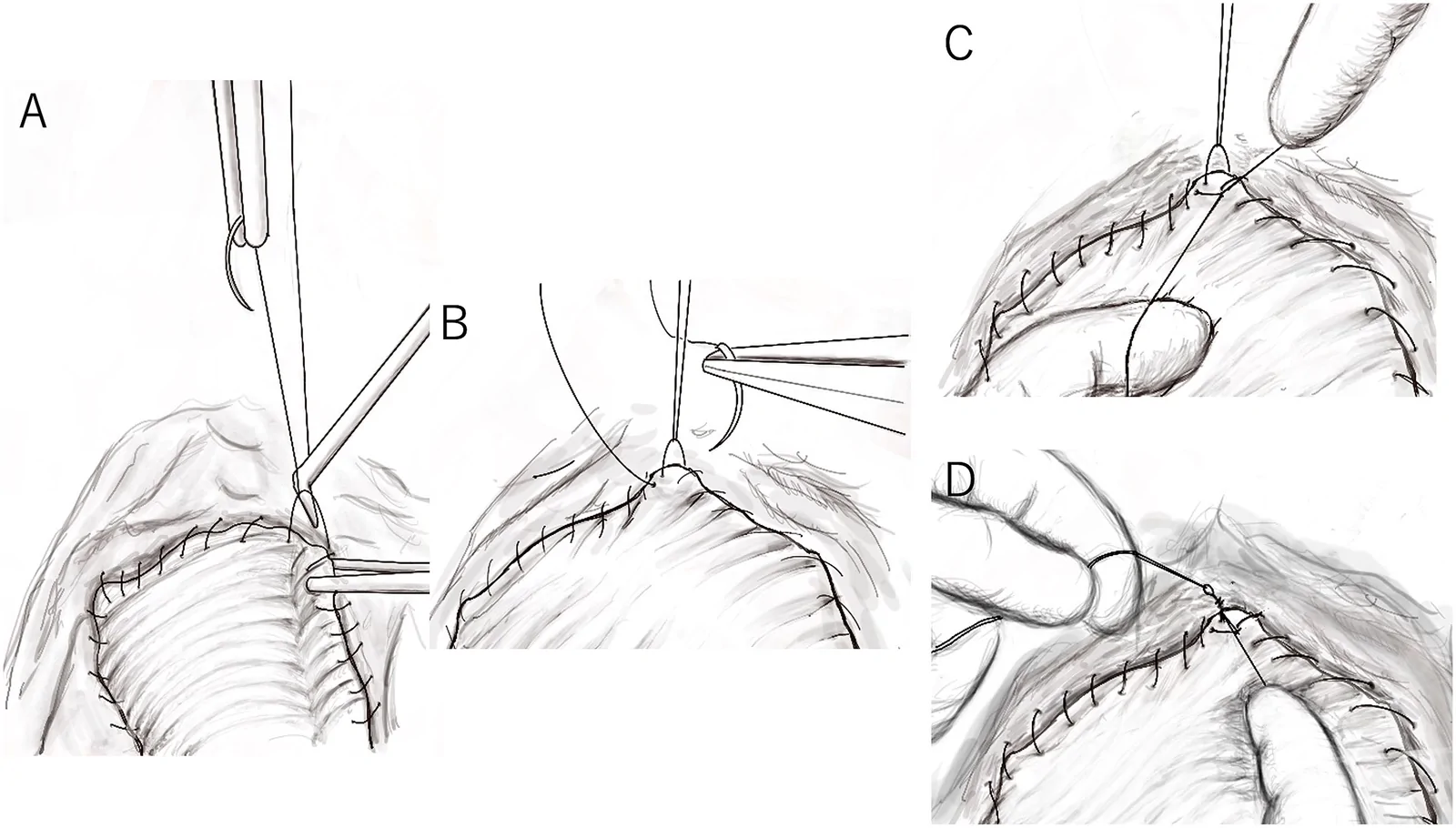
Re-tightening suture technique; (A) a selected point of the continuous suture line is lifted using a separate suture to expose the bleeding site; (B) an additional horizontal mattress suture using 4–0 polypropylene is placed at the same site; (C) the additional suture is gently tensioned and tied; (D) the redundant segment of the continuous suture is pulled and secured within the additional mattress suture, thereby re-tightening the original suture line.

Postoperative echocardiography showed no aortic regurgitation and a mean transvalvular pressure gradient of 4.2 mmHg. Computed tomography demonstrated satisfactory root geometry (Fig. 1E). The patient was discharged on postoperative Day 14.

## Discussion

VSRR preserves the native valve and avoids prosthetic valve-related complications, including anticoagulation-related bleeding associated with mechanical valves [2]. In this patient, recurrent skin bleeding caused by scratching due to poorly controlled atopic dermatitis supported the preference for a valve-sparing procedure. This consideration supported the choice of VSRR, but did not distinguish remodeling from re-implantation, because both preserve the native valve and generally avoid long-term anticoagulation.

Re-implantation provides robust annular and root stabilization but requires more extensive root dissection [3]. Remodeling preserves more physiological root dynamics and may require less dissection. In the present case, the cusps were suitable for preservation, regurgitation was mainly functional, and dense adhesions made extensive root dissection undesirable. We therefore selected remodeling with external annuloplasty. Objective measurements using a Hegar dilator and an SK-I template allowed reproducible control of annular size, graft configuration, and commissural height [4–6].

Bleeding from the proximal graft-to-native root suture line remains an important technical concern after remodeling. Comparative evidence is limited. In a recent propensity-matched study, re-exploration for bleeding was clinically more frequent after remodeling than after re-implantation (17.0% versus 4.5%), although the difference did not reach statistical significance after correction for multiple comparisons (*P* = .06) [7]. Thus, bleeding should be described as a technical concern rather than an established disadvantage inherent to remodeling.

Additional hemostatic sutures also present practical problems. Local plication or traction may alter the 3D geometry of the reconstructed root. A change in commissural relationships or cusp coaptation could theoretically cause new or residual aortic regurgitation. Adequate exposure is not always available, particularly near the coronary anastomoses. After restoration of cardiac activity, root motion and continuing bleeding may further obscure the operative field, making identification of the bleeding point and placement of an additional suture more difficult. Forceful exposure of tissue weakened by chronic dissection or inflammation may cause suture-line injury or tissue cut-through and worsen bleeding.

Our re-tightening technique addresses these problems by using a separate suture to lift a selected point of the continuous suture line. This maneuver locally exposes the bleeding point without requiring excessive traction on the surrounding tissue and facilitates placement of an additional horizontal mattress suture. The additional suture is then used to pull and secure the redundant segment of the original continuous suture, restoring tension along the suture line. The technique therefore combines exposure, reinforcement, and re-tensioning rather than simply adding another stitch. It may also reduce the need for continuous traction by the assistant and help avoid excessive tension on fragile native tissue.

This single case cannot establish that the technique reduces blood loss or preserves root geometry. Further experience and postoperative morphological assessment are required. Nevertheless, the re-tightening suture technique may provide a simple and reproducible option for controlling suture-line bleeding after root remodeling, particularly when exposure is limited.

## Supplementary Material

Video1__rjag954

## Data Availability

The data underlying this article will be shared on reasonable request to the corresponding author. Written informed consent for publication and sharing of relevant de-identified data was obtained from the patient.

## References

[1] Kunihara T, Neumann N, Kriechbaum SD et al. Aortic root remodeling leads to good valve stability in acute aortic dissection and preexistent root dilatation. J Thorac Cardiovasc Surg 2016;152:430–6. 10.1016/j.jtcvs.2016.03.076 27183883

[2] Iannacone EM, Lau C, Soletti G Jr et al. Aortic valve-sparing root replacement or Bentall? Ann Cardiothorac Surg 2023;12:168–78. 10.21037/acs-2022-avs1-170 PMC10248904 37304706

[3] Kunihara T . Current controversies in aortic valve-preserving surgery. J Cardiol 2023;81:119–30. 10.1016/j.jjcc.2022.10.006 36272618

[4] Kunihara T, Aicher D, Rodionycheva S et al. Preoperative aortic root geometry and postoperative cusp configuration primarily determine long-term outcome after valve-preserving aortic root repair. J Thorac Cardiovasc Surg 2012;143:1389–95. 10.1016/j.jtcvs.2011.07.036 21855091

[5] Saiydoun G, Saade S, Alghamdi M et al. A standardized step-by-step teaching approach of remodeling root replacement with external ring annuloplasty. J Thorac Cardiovasc Surg 2026;171:83–91. 10.1016/j.jtcvs.2025.04.035 40324750

[6] Kunihara T . Toward standardization of valve-sparing root replacement and annuloplasty. Gen Thorac Cardiovasc Surg 2018;66:685–91. 10.1007/s11748-018-1015-x 30225772

[7] Gofus J, Vojacek J, Karalko M et al. Aortic valve performance after remodelling versus reimplantation in a propensity-matched comparison. Eur J Cardiothorac Surg 2024;66:ezae234. 10.1093/ejcts/ezae234 39102871

